# Exome Sequencing Uncovers Genetic Drivers of Multiple Sclerosis in a Multiplex Family

**DOI:** 10.3390/genes16111311

**Published:** 2025-11-01

**Authors:** Carla Lintas, Simone Bonora, Anna Marabotti, Claudio Tabolacci, Maria Luisa Scattoni, Fioravante Capone, Mariagrazia Rossi, Vincenzo Di Lazzaro, Fiorella Gurrieri

**Affiliations:** 1 Research Unit of Medical Genetics, Department of Medicine, University Campus-Biomedico of Rome, 00128 Roma, Italy; 2 Operative Research Unit of Medical Genetics, Fondazione Policlinico Universitario Campus Bio-Medico, 00128 Roma, Italy; 3 Department of Chemistry and Biology “A. Zambelli”, University of Salerno, 84084 Fisciano, SA, Italy; sbonora@unisa.it (S.B.); amarabotti@unisa.it (A.M.); 4 Coordination and Promotion of Research, Istituto Superiore di Sanità, Viale Regina Elena 299, 00161 Rome, Italy; claudio.tabolacci@iss.it (C.T.);; 5 Operative Research Unit of Neurology, Fondazione Policlinico Universitario Campus Bio-Medico, 00128 Roma, Italy

**Keywords:** familial multiple sclerosis, whole exome sequencing, germinal, causative genes

## Abstract

**Background:** Multiple Sclerosis (MS) is a chronic, autoimmune, multifactorial, and complex disorder of the central nervous system (CNS), affecting more than 2 million individuals globally. Genome-wide association studies (GWAS) have explained only a small fraction of its high heritability, highlighting the need for alternative approaches to identify rare genetic variants that contribute to its etiology. To address this, we performed whole-exome sequencing (WES) in a multi-affected family. **Methods**: WES was performed in a MS multigenerational family comprising two affected sisters, their two healthy brothers, and one affected son. **Results:** Bioinformatics analysis identified 47 co-segregating rare variants. Three missense variants in genes involved in inflammation, autoimmunity, and demyelinization were identified as the most promising candidates: c.443 C>T, p.Pro148Leu in the *RTN4* gene, c.1678 T>G, p.Phe560Val in the *JAK2* gene, and c.3449 A>G, p.Tyr1150Cys in the *DUOX2* gene. Protein modeling and in silico tools suggest that the three selected variants may have a significant impact on protein function. **Conclusions:** We identified novel candidate genes for MS in a multiplex family, providing evidence for an oligogenic model of disease susceptibility. Further replication and functional studies are required to validate these preliminary results.

## 1. Introduction

Multiple Sclerosis (MS) is a chronic, autoimmune, multifactorial disorder of the central nervous system (CNS), characterized by its complex pathogenesis and affecting more than 2 million individuals globally [1]. It is one of the most prevalent inflammatory and neurodegenerative diseases among young adults, particularly in Western countries [2]. The clinical heterogeneity, variable penetrance, and familial clustering observed in MS suggest that an oligogenic and multifactorial model, involving the combined effect of multiple rare/common variants and environmental triggers, may better explain disease expression [3,4]. The clinical manifestations of MS vary depending on the anatomical location and severity of CNS lesions, which can involve the brain, spinal cord, or optic nerves. These lesions are primarily driven by the infiltration of peripheral immune cells—including CD4^+^ and CD8^+^ T lymphocytes, as well as B cells—through a compromised blood-brain barrier, ultimately leading to the targeting and destruction of myelin epitopes. Indeed, CD4^+^ T cell-mediated autoimmunity plays a central role in MS pathogenesis. Experimental autoimmune encephalomyelitis (EAE) has been extensively used as a murine model of MS, particularly due to its capacity to recapitulate CD4^+^ T cell-driven CNS inflammation, infiltration, and demyelination driven by Th1 and Th17 effector cells [5]. Recent immunological studies have identified cytokine-mediated pathways as critical modulators of neuroinflammation in MS. Within the interleukin-6 (IL-6) cytokine family, IL-11 has emerged as a key mediator linking innate and adaptive immunity. Zhang and colleagues [6] demonstrated that IL-11 drives the differentiation of encephalitogenic Th17 cells, characterized by IL-17A, GM-CSF, and IL-21 production, and promotes their migration across the blood–brain barrier through CCR6 and ICAM-1 upregulation. Building on these findings, Seyedsadr et al. [7] showed that IL-11 signaling activates the NLRP3 inflammasome in monocytes, enhancing IL-1β–dependent inflammatory cell trafficking to the central nervous system. Blockade of IL-11 with monoclonal antibodies markedly reduced demyelination and clinical severity in experimental autoimmune encephalomyelitis.

The familial aggregation observed in a substantial subset of MS cases supports a strong genetic contribution to disease susceptibility. Numerous family-based studies (e.g., [8]) have demonstrated that both common and rare genetic variants contribute to disease risk through complex interactions. Interestingly, sporadic MS cases have been reported to exhibit higher polygenic risk scores (PRS) compared to familial cases, suggesting a greater burden of rare, high-impact variants in familial forms of the disease.

Genome-wide association studies (GWAS) have identified more than 200 non-MHC loci, 32 loci within the major histocompatibility complex (MHC), and one X-linked locus associated with MS susceptibility [9]. HLA gene variants in HLA-DRB1, HLA-DR3, and HLA-DR4 are well-known determinants of MS susceptibility: the HLA-DRB1*15:01 allele is known to increase the risk of developing MS by over three times. However, these variants collectively explain only approximately 50% of the estimated heritability, indicating that rare variants with larger effect sizes—potentially identifiable through next-generation sequencing approaches—may account for a portion of the missing heritability. In this context, whole exome sequencing (WES) has been increasingly applied to multiplex families to uncover rare, deleterious variants potentially contributing to MS pathogenesis.

Environmental exposures are also recognized as key modulators of MS risk, often acting in concert with genetic predisposition. Notable environmental risk factors include cigarette smoking, obesity, hormonal influences (especially those related to puberty and female sex), low vitamin D levels, geographic latitude and related climate variables, night shift work, exposure to organic solvents, and infection with certain viruses—most notably the Epstein-Barr virus (EBV) [10].

The primary aim of this study is to identify novel MS-associated genes through WES in a multiplex Italian family affected by MS. In parallel, we conducted a comprehensive review of the literature concerning genetic studies in MS, with the goal of compiling an updated and curated list of candidate genes that may have diagnostic and clinical relevance.

## 2. Materials and Methods

### 2.1. Whole Exome Sequencing

**Figure 1:**
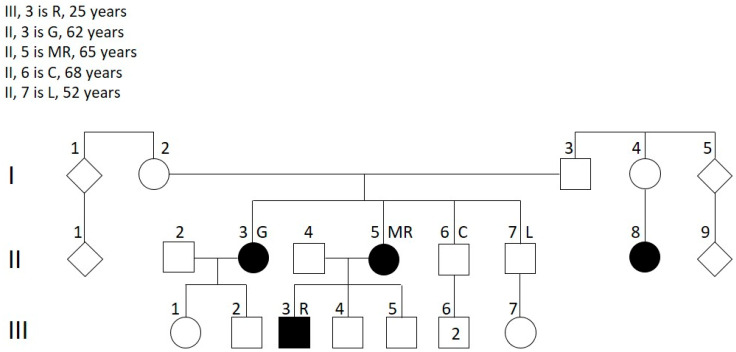
Three-generation family pedigree; whole exome sequencing was performed in II3, II5, II6, and III3. Black symbols represent affected individuals. Square and circle represent male and female, respectively.

A multigenerational family was referred for genetic counseling by the neurologist because of a high incidence of MS among its members (Figure 1). The affected individuals included two sisters (II3, 62 years old; II5, 65 years old), their cousin (II8), and the son of one of the sisters (III3, 25 years old).

Whole exome sequencing (WES) was performed on the three probands (II3, II5, and III3) and in one of their two unaffected brothers (II6), achieving an average coverage of 60× on an Illumina platform (Figure 1). Genomic DNA was extracted from peripheral blood leukocytes. The bioinformatics analysis was performed using the online platform Galaxy [11]. FastQ files were aligned to the human reference genome (Human GRCh37/hg19) with the Burrows-Wheeler Aligner [12], duplicates were removed, and variant calling was performed using FreeBayes.

**Figure 2:**
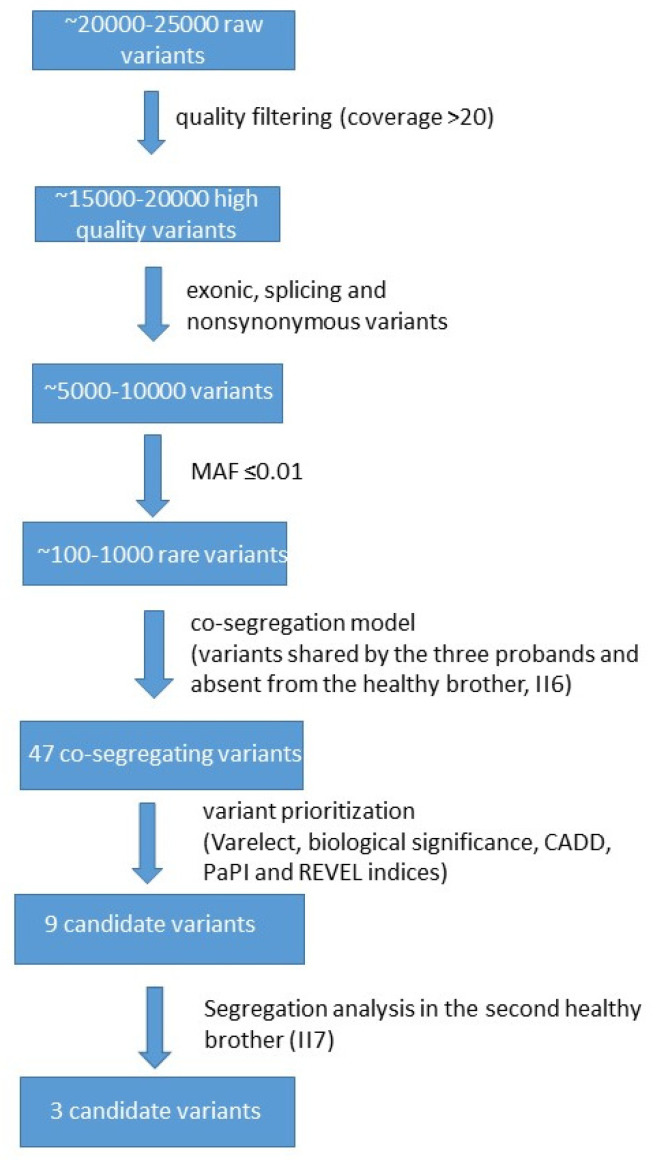
Bioinformatic workflow adopted in this study.

In our WES analysis (Figure 2), we ensured high data quality with over 90% of bases exceeding Q30 and coverage uniformity above 80%, supporting reliable and consistent variant detection across the exome. Each individual initially showed ~20,000–25,000 raw variants, which were reduced to ~15,000–20,000 after quality filtering (e.g., depth, geno-type quality, call rate). Following functional annotation (e.g., selecting exonic, splice-site, nonsynonymous, LoF variants), the number decreased to ~5000–10,000. Subsequent frequency filtering (e.g., MAF < 0.01 or absent in population databases like gnomAD) yielded ~100–1000 rare variants. Applying the co-segregation model further narrowed this to 47 variants (Table S1). These variants were shared by the three probands and were absent from the healthy brother.

The enGenome-eVai (CE-IVD) software (Version v 3.7) was used for variant annotation (https://www.engenome.com, accessed on 10 January 2025). We filtered for variants with the following parameters: (a) variant frequency lower than 1% using several databases as GnomAD, ExAC, 1000 Genomes, Exome Sequencing Project; (b) type of variants: missense, stop, frameshift, splicing, indels; (c) Coverage ≥ 20; (d) Variant Allele Frequency ≥ 0.4 (number of reads of alternate allele/total number of reads given by the sum of the reference and the alternate alleles); (e) exclusion of benign and likely benign variants classified according to the American College of Medical Genetics (ACMG) guidelines [13]. After filtering variants with e-Vai, variants were prioritized according to their pathogenicity using ACMG guidelines and the online NGS phenotyper tool VarElect (https://varelect.genecards.org/about/, accessed on 10 January 2025) [14]. The following keywords were used for VarElect prioritization analysis: “demyelinization” OR “inflammation” OR “autoimmunity”. To identify the most promising candidate genes for MS, we evaluated each variant based on its predicted pathogenicity (primarily assessed using enGenome-eVai) and the involvement of the corresponding gene in MS pathogenesis, as determined by VarElect. This evaluation process allowed us to create a final list of nine best candidate gene variants. In silico prediction tools for missense variants were accessed via Varsome, and CADD, REVEL, and PaPI scores were used to assess potential pathogenicity (https://varsome.com/, accessed on 10 January 2025). Variant frequencies were obtained using the GnomAD database (https://gnomad.broadinstitute.org/, accessed on 10 January 2025).

We then performed segregation analysis for the best candidate variants in the other healthy brother (II7) of the two affected sisters (II3 and II5). A total of nine variants were tested, as two genes had two hits each. The only variants that were absent from both healthy brothers were those in the *RTN4*, *JAK2*, and *DUOX2* genes. Segregation analysis of these three variants was also performed in selected individuals of the third generation (Figure 1): III1 (30 years old), III2 (24 years old), III4 (24 years old), and III5 (20 years old). Unfortunately, individual II8 was not available for segregation analysis, and individuals III6 and III7 were not informative because their fathers did not carry any of the three variants. In addition to rare variants, we also filtered exomes for the most common polymorphisms (frequency higher than 0.01) associated with MS (International Multiple Sclerosis Genetics Consortium, 2007) and for the HLA-DRB1*15:01 allele.

The study was approved by the local ethics committee (Institutional Review Board approval no. 04.21) and conducted in accordance with the Declaration of Helsinki. Written informed consent was obtained from all participants.

### 2.2. Bioinformatics Analysis for Protein Structure

The crystallographic structure used for various analyses on both the wild-type and mutant forms of the protein JAK2 to identify potential differences is the one with the PDB code 6BS0, retrieved from the RCSB Protein Data Bank (PDB) [15] (https://www.rcsb.org/structure/6BS0, accessed on 24 July 2025). This structure was determined by X-ray diffraction, with a resolution of 1.54 Å. However, structure 6BS0 lacks the side chains of several residues. To address this issue, we remodeled the sequence using SwissModel (https://swissmodel.expasy.org, accessed on 24 July 2025) [16], using the original structure as a template. This approach allowed us to preserve the backbone and existing side chain conformations while reconstructing the missing side chains.

For DUOX2, only the model of the protein structure obtained by AlphaFold 2 and included in the AlphaFold DB (code: AF-Q9NRD8-F1) [17] is available. Most portions of this model show a good confidence (pLDDT > 70), and in particular, mutation p.Tyr1150Cys is located in the core of the protein, which is modeled with a very high reliability (pLDDT > 90). Therefore, this model can be considered a reliable starting point for following structural investigations.

We applied the Mutate Model procedure derived from the program MODELLER for comparative protein modeling [18] to generate the structural models of the two proteins carrying the p.Phe560Val mutation (for JAK2) and p.Tyr1150Cys mutation (for DUOX2). The resulting models were assessed using QMEANDisCo [19] and ProSAWeb [20] using default parameters; a comparison between wild-type and mutant structures was also made by using the Structure Assessment service (https://swissmodel.expasy.org/assess, accessed on 24 July 2025) [21], using default parameters. A range of analyses was then conducted on both the wild-type and mutant structures using a combination of web-based tools and locally installed software. In particular, residue–residue interactions were analyzed using RING 4.0—Residue Interaction Network Generator (http://protein.bio.unipd.it/ring/, accessed on 24 July 2025), secondary structures were assessed with DSSP [22], variation in solvent accessibility were evaluated by NACCESS [23], and the impact of mutation on protein stability was predicted by using three different web tools: DynaMut2 (http://biosig.unimelb.edu.au/dynamut2/, accessed on 24 July 2025), DUET (http://biosig.unimelb.edu.au/duet/, accessed on 24 July 2025), and INPS-MD (https://inpsmd.biocomp.unibo.it/, accessed on 24 July 2025).

No in silico structural analysis was performed for the RTN4 variant, as no reliable structure was available for modeling. In fact, the model available for this protein, predicted by AlphaFold2 (https://alphafold.ebi.ac.uk/entry/Q9NQC3, accessed on 24 July 2025), shows a largely unreliable and most likely disordered structure, making it impossible to make reliable predictions about the effect of mutations at the level of protein structure and function.

### 2.3. Patient Clinical Presentation

Detailed clinical history was available for patients II3 and III3 only.

**Patient II3**: She is a 62-year-old female (Figure 1). The disease onset occurred at age 27 with an episode of diplopia, which was treated with corticosteroids. In the following years, the patient experienced several relapses, particularly after her two pregnancies. Following the second pregnancy, she had a significant relapse characterized by a right-sided motor hemisyndrome, during which she underwent a brain MRI revealed demyelinating lesions. A diagnosis of MS was established, and treatment with Betaferon was initiated and continued until 2009, when it was discontinued due to flu-like symptoms.

Between 2012 and 2014, she started therapy with Gilenya, which was subsequently discontinued after the detection of a lesion on the hemilingual region; histological examination confirmed it to be benign. She is currently undergoing treatment with Ofatumumab. She has been using a wheelchair for approximately 12 years. She reports urinary urgency with occasional incontinence and episodes of dysphagia for liquids. She is currently undergoing physiokinesitherapy (PKT). She reports previous use of Lioresal and Sativex, both discontinued due to excessive drowsiness.

Neurological Examination (EON): during neurological examination, the patient was alert, cooperative, and oriented. Speech was dysarthric but remained intelligible. A mild flattening of the right nasolabial fold was observed. There was right brachio-crural hypoesthesia.

In the upper limbs, spasticity was present in the right upper limb, which was held in a fixed flexed posture. The patient was able to lift the left upper limb but was unable to maintain it in an anti-gravity position.

In the lower limbs, there was plegia of the right lower limb and severe paresis of the left lower limb.

The patient was able to attain an upright position with bilateral support but was unable to ambulate.

The Expanded Disability Status Scale (EDSS) score was 8.5.

**Patient III3:** He is a 25-year-old male (Figure 1) and the nephew of patient II3. The patient reported the onset of gait instability approximately 5 years ago. In 2021, he experienced an undefined episode of visual disturbance. Brain and spinal cord MRI revealed multiple demyelinating lesions. In 2022, a new contrast-enhancing supratentorial lesion was identified and treated with oral corticosteroids (Deltacortene). Subsequently, he experienced gradual and progressive worsening of gait and a decline in motor autonomy without clearly defined clinical relapses at onset or during follow-up. MRI performed on 30 June, 2023, demonstrated multiple demyelinating lesions in both supratentorial and infratentorial regions, as well as spinal cord involvement at levels C2–D1, D3–D4, D6–D8, and from D9 to the apex of the conus medullaris. The STRATIFY fall risk index was positive (score 3.85). Ocrelizumab therapy was initiated on 23 August, 2022. The patient undergoes physiokinesitherapy three times per week and is followed at a hospital Multiple Sclerosis Center in south of Italy. Current home treatment includes Expose, Folina, and Ocrelizumab. Neurological examination revealed mild leftward deviation of the protruded tongue, bilateral internuclear ophthalmoplegia without diplopia, bilateral kinetic tremor on finger-to-nose testing (more pronounced on the left), no drift on anti-gravity maneuvers, preserved bilateral hand grip, hyperactive deep tendon reflexes (predominantly on the left), spastic-ataxic gait, positive Romberg sign, preserved graphesthesia, ambulatory capacity of 200 m without stopping, EDSS score of 5.0, nasal voice, and fissuring of the tongue.

**Patient II5**: A 65-year-old female, mother of Patient III3. Only limited clinical information is available, as reported by her eldest brother (II6). The clinical presentation and disease course closely resembled those of her affected sister (II3). The first symptoms appeared in her late 20s, and the diagnosis was established after her first pregnancy, at the age of 36.

### 2.4. Literature Review

WES studies related to MS multiplex families were retrieved from the literature using Pubmed (https://pubmed.ncbi.nlm.nih.gov/, accessed on 20 March 2025) by introducing the following search string: “multiple sclerosis[Title/Abstract] AND (whole exome sequencing[Title/Abstract] OR exome sequencing[Title/Abstract]) AND (familial[Title/Abstract] OR family-based[Title/Abstract] OR families[Title/Abstract])”. The studies identified through this literature search were selected by excluding those related to other pathologies or comorbidities and those that used other genomic technologies.

## 3. Results

We first checked for common polymorphisms associated with MS, filtering for well-known MS-associated SNPs [24], including the HLA-DRB1*15:01 allele, which is considered the most significant genetic risk factor in the Northern European population. This allele increases the risk of MS by over three times. None of them was found in our patients.

Forty-seven rare variants were shared by the three probands and absent from one of the two healthy brothers (Figure 1; Table S1). Each gene variant was individually evaluated based on:

- Minor allele frequency (MAF) and number of homozygotes using the GnomAD v2.1.1 database (https://gnomad.broadinstitute.org/, accessed on 10 January 2025);
- Predicted functional impact using in silico tools and integrated scores including CADD, REVEL, and PaPI indices;
- Biological relevance to processes like “autoimmunity”, “inflammation”, and “demyelinization” assessed through literature review and the NGS phenotyper tool VarElect;
- Tissue-specific expression profiles, utilizing data from the GTEx database (https://www.gtexportal.org/home/aboutAdultGtex, accessed on 10 January 2025).

We then performed segregation analysis in the second healthy brother for the best nine selected candidates. Three final variants shared by the probands (II3, II5, and III3) and absent from the two healthy brothers (II6 and II7) were finally selected. Table 1 summarizes the molecular characteristics of the *RTN4*, *JAK2,* and *DUOX2* variants.

The first variant is c.443 C>T, p.Pro148Leu in the *RTN4* gene. No diseases are currently associated with the *RTN4* gene (OMIM* 604475) on the database of genetic disease OMIM (https://www.omim.org/, accessed on 10 January 2025). In the database, ClinVar is reported as only VUS or benign variants not associated with a specific phenotype. The variant *RTN4* c.443 C>T, p.Pro148Leu is not present in ClinVar and is absent from GnomAD. The affected amino acid p.Pro148Leu falls within the proline-rich disordered domain of the protein located close to the amino-terminus, and, according to the database UniProt (https://www.uniprot.org/, accessed on 10 January 2025), this amino acid falls within the domain responsible for the inhibitory effect on neurite outgrowth and the spreading of neurons. No in silico structural analysis was performed for this variant as no reliable structure was available, since most parts of the protein structure (including the one in which the mutation falls) are totally disordered, as it is possible to see from the model predicted by AlphaFold2 and publicly available through AlphaFold Database (https://alphafold.ebi.ac.uk/entry/Q9NQC3, accessed on 24 July 2025). The CADD index is rather high, and a “quite likely deleterious” impact is predicted (Table 1). In addition, the conservation index is moderate (see Table 1), suggesting that the amino acid Pro148 is conserved across different species and that the change to Leu could affect the protein function. In silico predictions are conflicting: 3 pathogenetic, 4 VUS, and 21 benign variants have been reported (Table 1).

**Figure 3:**
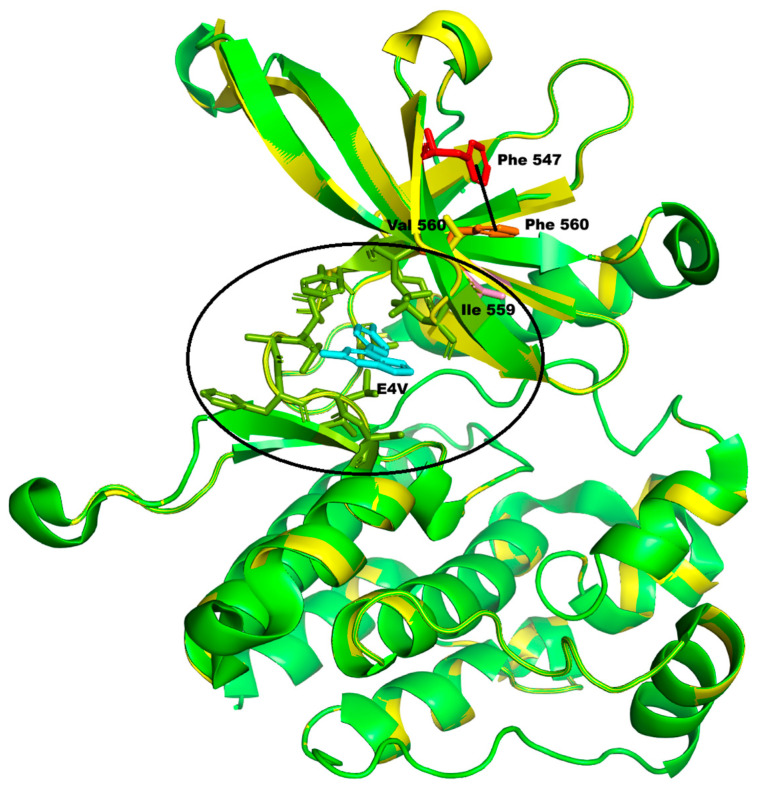
Molecular changes associated with the *JAK2* c.1678 T>G, Phe560Val variant. In the structure of the wild-type protein (green), Phe 560 (orange) interacts with Phe 547 (red), forming a π–π stacking interaction (black line) that could stabilize the central β-sheet. The replacement of Phe560 with Val determines the loss of this interaction in the structure of the mutant (in yellow). Moreover, Phe560 is adjacent to Ile559 (pink), which is part of the binding pocket (olive green, in the circle) of the inhibitory molecule 4-(5-aminopyrazin-2-yl)-1H-pyrrolo [2,3-b]pyridin-6-amine (E4V, in cyan) present in the selected structure (PDB file: 6BS0). The replacement of Phe560 with Val may perturb this binding pocket.

The second variant, c.1678 T>G, p.Phe560Val, is located in the *JAK2* gene. The *JAK2* gene encodes Janus Kinase 2, a non-receptor tyrosine kinase that plays a pivotal role in the signaling pathways of various cytokines and growth factors. In the OMIM database, the *JAK2* gene (OMIM*147796) is associated with several haematological malignancies, but only at the somatic level. The CADD, REVEL, and PaPI indices (Table 1) are all relatively strong, suggesting that the p.Phe560Val change may adversely affect protein function. Also, the high conservation index (7.684) is in favour of a likely pathogenic effect. We have modeled the mutation selecting the structure of wild-type protein available in PDB code 6BS0, which has an excellent overall quality as per the wwPDB validation report (https://files.rcsb.org/validation/view/6bs0_full_validation.pdf, accessed on 24 July 2025). The resulting model has an overall QMEANDisCo confidence score of 0.91 ± 0.05 and a local QMEANDisCo confidence score of 0.93 on position 560. The Ramachandran plot shows 98.89% of the residues (including position 560) in the favored zones, with no outliers. Finally, the overall ProSA Z-score is −7.50, confirming the good model quality. The Phe560Val change falls within the protein kinase 1 domain (amino acids 545-809) of the protein. Indeed, the INPS-MD and DUET softwares predict that the change will cause protein destabilization (Figure 3).

Although the secondary structure containing Phe560 is not altered by the variation, the solvent accessibility of Val560 is reduced, while that of two spatially close residues (Lys558 and Ser550) increases, varying the polarity of that zone. The replacement of Phe560 with Val also causes the loss of some van der Waals interactions with Ser550 and Leu580, as well as one π–π stacking interaction (Phe560–Phe547), which may play a role in stabilizing the central β-sheet of the domain (Figure 3, dotted line). Moreover, this residue is adjacent to Ile559, which in the selected structure is part of the binding site (indicated with the circle in Figure 3) of an inhibitory molecule of the protein’s activity, and consequently, the p.Phe560Val substitution may also indirectly perturb this protein binding pocket.

**Figure 4:**
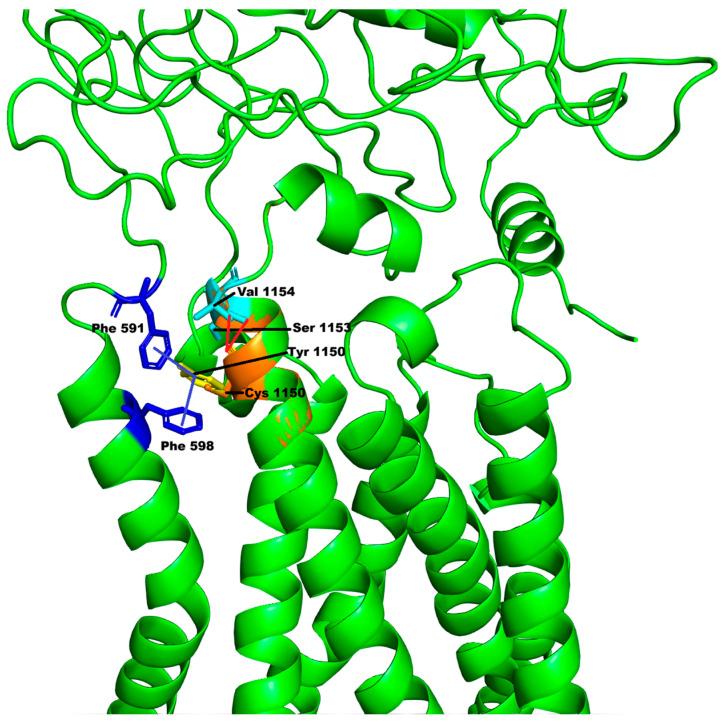
Molecular changes associated with the *DUOX2* c.3449 A>G, p.Tyr1150Cys variant. In the wild-type protein (green), Tyr1150 (yellow) interacts with Phe 591 and Phe 598 (blue), forming π–π stacking interactions (blue lines), and with Ser 1153 and Val 1154 (cyan), with hydrogen bonds (red lines). The replacement of Tyr1150 with Cys (orange) determines the loss of all these interactions in the mutant protein. The upper part of the protein (more disordered) represents the predicted peroxidase-like domain, to which Phe 591 belongs, whereas the lower part of the protein (with the alpha-helix bundle) represents the ferric reductase-like transmembrane component.

The third variant, c.3449 A>G, p.Tyr1150Cys in the *DUOX2* gene, is correlated in the OMIM database with thyroid dyshormonogenesis 6 (OMIM# 607200). The variant (Table 1) has already been reported twice as a VUS in ClinVar and has high CADD and PaPI scores (26.2 and 1, respectively). The *DUOX2* encoded protein belongs to the NOX (NADPH oxidase) family responsible for reactive oxygen species generation that under certain conditions can lead to oxidative stress. We have modelled the mutation starting from the model available in the AlphaFold DB (code: AF-Q9NRD8-F1). The resulting model has an overall QMEANDisCo confidence score of 0.78 ± 0.05 and a local QMEANDisCo confidence score of 0.89 on position 1150. The Ramachandran plot shows 94.95% of the residues (including position 1150) in the favored zones. Finally, the overall ProSA Z-score is −13.11, confirming the good model quality. Protein structure analysis for this variant is reported in Figure 4.

Tyr1150 is a residue located in one of the long α-helices forming the ferric reductase-like transmembrane component of the DUOX2 protein (represented as the lower part of Figure 4). The variation p.Tyr1150Cys is not predicted to affect the secondary structures of the protein, and the solvent accessibility of Cys1150 is reduced only because of the smaller size of the mutated amino acid. However, the replacement of Tyr1150 with Cys causes the loss of π–π stacking interactions (highlighted in Figure 4 with the blue lines) with Phe591 (belonging to the predicted peroxidase-like domain, represented as the upper part of Figure 4) and Phe598, as well as the loss of hydrogen bonds with residues Ser1153 and Val1154 (Figure 4, dotted cyan lines). Therefore, this could produce destabilization of the protein structure, as confirmed by the analysis of the predictors of stability DUET and INPS-MD, which both predict a destabilizing impact of the mutation on the protein structure, whereas DynaMut predicted a slight stabilizing effect.

Segregation analysis of the three variants was performed in individuals of the third generation (Figure 1; Table 2): III1 (30 years old), III2 (24 years old), III4 (24 years old), and III5 (20 years old). The c.3449 A>G variant in the *DUOX2* gene was present in III1, III4, and III5 and absent from the III2 individual. The c.443 C>T variant in the *RTN4* gene was present only in the III1 individual, whereas the c.1678 T>G variant in the *JAK2* gene was inherited by III1 and III5. Individual III1, who is the only one to have inherited the three variants, reported undergoing a non-contrast MRI following tingling in the upper limbs and numbness in the lower limbs. At present, she does not have an MS diagnosis.

## 4. Discussion

Multiple sclerosis is characterized by marked clinical heterogeneity and incomplete penetrance, both in terms of symptom expression and age of onset, reflecting its multifactorial and oligogenic nature [25,26]. These features may explain why individual III-1 currently exhibits only a subset of the disease manifestations. Such variability is consistent with an oligogenic and multifactorial model, in which the combined effect of multiple rare variants, together with environmental and epigenetic modifiers, influences disease expressivity and penetrance within affected families [25,26]. Next-generation sequencing (NGS) technologies have significantly enhanced our understanding of both Mendelian and complex diseases. Among these approaches, whole-exome sequencing (WES)—that targets all protein-coding regions—offers a cost-effective and efficient method for identifying rare and inherited pathogenic variants that may be missed by targeted gene panels [27]. In familial cases like the one described here, WES enables the identification of novel disease-associated genes and provides insights into inheritance patterns and the molecular mechanisms driving disease progression.

We compared our WES findings from a multiplex family affected by multiple sclerosis (MS) with previously published data (Table 3).

As summarized, prior studies have identified various candidate genes and implicated several key pathways—such as vitamin D metabolism, TNF signaling, and broader immunological networks—in the etiology of familial MS [35,36,38,40]. These findings underscore the complex pathogenesis and multifactorial hereditary architecture of MS. Notably, Horjus and colleagues [31] reported 12 rare exonic variants that co-segregated with MS in at least one family, involving genes related to myelination/demyelination and autoimmune regulation, supporting an integrated molecular model of MS pathophysiology. Similarly, Everest et al. [8] showed that individuals with familial MS carry not only rare variants but also exhibit an increased burden of common MS-associated alleles, suggesting a combined role of rare and common variants in disease risk. In one of the largest family-based WES studies to date, Büyükgöl et al. [28] identified novel rare variants associated with extracellular matrix organization and hemidesmosome assembly—pathways potentially contributing to familial MS susceptibility. Notably, a rare variant in the *DUOX2* gene, reported in their cohort [28], also emerged as a candidate in our family (see Table 2).

Through WES, we identified three rare missense variants in MS-related genes in the affected individuals from a multigenerational multiplex family. Our three candidate genes are all involved in inflammation, immunity, and oxidative stress pathways, similar to the genes reported in the other WES studies (Table 3). These variants may act “synergistically and/or in combination with shared environmental exposures” to contribute to MS onset and progression in our family. All affected family members reside in the same village in southern Italy, potentially increasing the influence of common environmental risk factors.

We hypothesize that these three missense variants act via gain-of-function mechanisms:

- The c.443 C>T, p.Pro148Leu variant in *RTN4*, encoding Nogo-A, may enhance neuroinflammation and inhibit myelin regeneration in active lesions.
- The c.1678 T>G, p.Phe560Val variant in *JAK2* may result in constitutive activation of the JAK2/STAT3-signaling pathway, leading to excessive Th1/Th17 activation, oxidative stress, and autoimmune targeting of myelin basic protein.
- The c.3449 A>G, p.Tyr1150Cys variant in *DUOX2* may increase the production of reactive oxygen species (ROS), promoting oxidative stress, a known contributor to cerebrovascular injury in MS and other neurodegenerative diseases.

All three genes are expressed in the central nervous system (CNS), as confirmed by the GTEx database (https://www.gtexportal.org/home/, accessed on 10 October 2025).

The *RTN4* encodes three isoforms, sharing a conserved C-terminal domain but differing in their N-terminal domains due to alternative splicing [41]. Among them, the Nogo-A isoform, predominantly expressed in oligodendrocytes, neurons, and microglia across brain regions, is the most potent myelin-associated inhibitor of axonal regeneration and plasticity. In the adult CNS, Nogo-A is mainly localized to the inner and outer myelin membrane, functioning as a long (1192 amino acids) transmembrane protein residing in the plasma and endoplasmic reticulum membranes. Nogo-A interacts with its receptor LINGO-1 to regulate oligodendrocyte differentiation, myelin sheath formation, and axonal growth. Deletion of either Nogo-A or its receptor delays these processes in neonatal mice. LINGO-1 negatively regulates myelination by inhibiting the receptor tyrosine kinase ErbB2, thereby preventing its translocation to lipid rafts and subsequent phosphorylation. In addition, Nogo-A contributes to neuroinflammation through activation of NgR signaling, which promotes pro-inflammatory cytokine expression and impairs cell adhesion and migration. Elevated Nogo-A levels have been observed in demyelinated lesions in MS patients [42]. Preclinical models using anti-Nogo-A antibodies or siRNA have demonstrated reduced inflammation and demyelination along with enhanced axonal repair and plasticity. Phase I clinical trials with anti-Nogo-A antibodies in demyelinating diseases show promise as therapeutic interventions.

JAK2, a member of the Janus kinase family, is a key component of the JAK-STAT-signaling cascade. Upon cytokine or growth factor binding, JAK2 undergoes phosphorylation and, in turn, phosphorylates STAT proteins, which then translocate to the nucleus to regulate gene expression. JAK2 plays a crucial role in immune regulation, inflammation, and hematopoiesis, and its dysregulation is implicated in several hematologic malignancies. The JAK2/STAT3 axis is particularly relevant in the differentiation of Th17 cells from naïve CD4^+^ T cells, a process essential to MS pathogenesis. Recent studies have demonstrated that pharmacological inhibition of this pathway (e.g., via AG490) alleviates the severity of EAE, a widely used MS model, by reducing Th17 cell differentiation and restoring autophagy [43].

DUOX2, a member of the NOX family of NADPH oxidases, is expressed in neurons, glial cells, and cerebrovascular endothelial cells. Enzymes of this family are recognized as major contributors to oxidative stress-related neurodegeneration, including in MS [44]. Increased ROS production by DUOX2 may exacerbate oxidative damage, promote inflammation, and contribute to blood–brain barrier disruption—hallmarks of MS pathology.

## 5. Conclusions

In summary, this study has identified three novel top candidate genes potentially associated with MS, co-segregating in a multigenerational family comprising four affected individuals. Future directions include: (i) functional validation of the identified co-segregating variants (Table 1); (ii) extension of WES analysis to additional multiplex MS families, with the aim of expanding the list of candidate genes or confirming those already identified; (iii) implementation of case–control association studies.

Following the development of an expanded targeted gene panel—integrating candidate genes reported in our study and others—we aim to screen a larger cohort to further clarify the genetic basis of MS.

We acknowledge several limitations of the current study, including the small sample size derived from a single pedigree and the absence of replication in independent MS cohorts. Furthermore, transcriptomic or proteomic data to support the functional relevance of the identified variants are currently lacking. Despite these constraints, this study constitutes a meaningful contribution to the identification of rare, potentially pathogenic variants through a family-based WES approach, underscoring the value of rare variant discovery in elucidating the genetic architecture of MS. In addition, the proposed genes provide insight into biological pathways (inflammation, ROS metabolism, myelin development) impaired in MS, representing potential targets for therapeutic intervention. All the identified variants are currently classified as VUS, and, therefore, no pathogenic role can be inferred at this stage. Further functional studies, as well as analyses in independent cohorts, will be required to validate their potential contribution to disease susceptibility.

Furthermore, the three prioritized variants identified in this study—RTN4 p.Pro148Leu, JAK2 p.Phe560Val, and DUOX2 p.Tyr1150Cys—highlight distinct yet potentially convergent therapeutic avenues in MS.

The RTN4 substitution may enhance Nogo-A signaling, reinforcing neurite outgrowth inhibition and impaired remyelination. Thus, anti-Nogo-A or anti-LINGO-1 monoclonal antibodies emerge as rational candidates to promote axonal plasticity and remyelination in genetically defined subgroups.

Conversely, the JAK2 missense change affects the kinase domain and may result in aberrant JAK2/STAT3 pathway activation, which sustains Th17 differentiation and neuroinflammation. Selective JAK2/STAT inhibitors could, therefore, represent genotype-guided immunomodulatory interventions, with phospho-STAT3 and Th17 cytokine profiling as potential pharmacodynamic biomarkers.

Finally, the DUOX2 variant may alter NADPH oxidase activity and reactive oxygen species (ROS) homeostasis, contributing to oxidative damage and blood–brain barrier dysfunction. NOX/DUOX-targeted inhibitors or antioxidant therapies might attenuate oxidative stress in carriers of this variant. Collectively, these findings support a precision medicine framework in MS, in which genetic stratification may inform mechanism-based therapeutic strategies integrating remyelination enhancement, immune signaling modulation, and oxidative stress control. Functional validation and genotype-enriched clinical studies will be essential to translate these hypotheses into targeted therapeutic development.

In conclusion, while the identified variants are putative and require further experimental validation through functional studies, we believe that our research constitutes an initial step toward the identification of gene variants that may contribute to increased susceptibility to MS.

## Figures and Tables

**Table 1:** Summary of the evaluation of the three strongest candidate variants.

Gene	Variant	MAF (Gnomad)	CADD Score	REVEL Score	PaPI Score	In Silico Algorithms	Conservation Score (phyloP100)	ClinVar	ACMG Classification
*RTN4*	NM_020532.5 c.443C>T p.Pro148Leu	absent	23.6 *	0.112	0.065	3P/4VUS/21B	4.209 (vus)	not reported	VUS (Class3)
*JAK2*	NM_004972 c.1678T>G p.Phe560Val	absent	28.2 *	0.666 *	1 *	9P/9VUS/3B	7.684 *	not reported	VUS (Class3)
*DUOX2*	NM_001363711.2 c.3449 A>G, p.Tyr1150Cys	0.00597%	26.6 *	0.532	1 *	3 VUS	5.18 (vus)	VUS (2 entries)	VUS (Class 3)

* = pathogenetic; vus = variant of unknown clinical significance. MAF = Minor Allele Frequency as reported in the database GnomAD including all genomic ancestry groups; phyloP100 = conservation score; In silico tools = number of algorithms reprting the variant as P = Pathogenetic; VUS = Variant of Unknown Significance; B/LB = Benign/Likely Benign; REVEL (Rare Exome Variant Ensemble Learner) index is a computational score used in genetics to predict the pathogenicity; CADD (Combined Annotation Dependent Depletion) score is a computational tool used in genetics to estimate the deleteriousness of genetic variants across the genome; PaPI (Pathogenicity Assessment of Pathogenicity Indicators) score is a computational metric used to evaluate the pathogenic potential of genetic variants, particularly rare ones; commonly used thresholds include CADD > 20, REVEL > 0.5–0.7, PaPI > 0.5. ACMG = American College of Medical Genetics.

**Table 2:** Segregation analysis for the three variants in individuals belonging to the II and the III generations.

ID	AGE (ys)	Phenotype	RTN4: c.443 C>T	JAK2: c.1678 T>G	DUOX2: c.3449 A>G
II3	62	affected	+	+	+
II5	65	affected	+	+	+
II6	68	healthy	−	−	−
II7	52	healthy	−	−	−
III1	30	some symptoms	+	+	+
III2	24	healthy	−	−	−
III3	25	affected	+	+	+
III4	24	healthy	−	−	+
III5	20	healthy	−	+	+

+ = presence of the variant; − = absence of the variant.

**Table 3:** WES studies in MS multiplex families.

Number of Families	Individuals WES Performed	Co-Segregated and Common-Risk Genes in Multiple Families	Reference
59	215	*ANKRD44*, *LAMB1*, *KRT14*, *HLA-DRB1*, *PALLD*, *RLF*, *SPI1*, *NLRC5*, *MICAL2*, *FZD9*, *SEZ6L*, *DST*, *FBN3*, *AHNAK*, *TTN*, *TDG*, *SPDL1*, *CD93*, *DUOX2*, *KRT14*, *ZNF717*, *CD109*, *ITPR1*, *ITGB4*	[28]
21	127	*P2RX4*, *P2RX7*, *CAMKK2*,	[29]
28	161	*UBR2*	[30]
7	41	*SZT2*, *TCEANC2*, *CYB5RL*, *GREB1*, *ARAP2*, *C5orf22*, *SRFBP1*, *PCDHAC1*, *PCDHB7*, *PCDHB16*, *PCDHB9*, *PCDHB10*, *PCDHB12*, *PCDH1*, *LRWD1*, *CDHR3*, *ENPP2*, *COL14A1*, *LRRC6*, *MASTL*, *CHD8*, *MYO5B*, *SERPINB13*, *SERPINB10*, *FSD1*, *SLC25A23*, *DENND1C*, *DNMT1*, *NOTCH3*, *NWD1*, *MUC17*	[8]
9	31	*MBP*, *PLK1*, *MECP2*, *MTMR7*, *TOX3*, *CPT1A*, *SORCS1*, *TRIM66*, *ITPR3*, *TTC28*, *CACNA1F*, *PRAM1*	[31]
5	24	*CUL9*, *ATP9A*, *TTBK1*, *PPP2R5D*, *MYO16*	[32]
3	16	*POLD2*, *POLM*, *TSN3*, *NBPF1*	[33]
4	14	*DPH3*, *INPP4B*, *MBP*, *ThyN1*, *NFAT5*, *TTC21A*	[34]
19	116	TNF-α receptors and ligands	[35]
15	94	Vitamin D signaling pathway	[36]
34	132	*PLAU*, *MASP1*, *C2*, *NLRP12*, *UBR2*, *CTNNA3*, *NFATC2*, *RNF213*, *NCOA3*, *KCNG4*, *SLC24A1*, *SLC8B1*	[37]
1	4	*FKBP6*	[38]
1	4	*TYK2*	[39]
43	43	*CBLB*, *IL7R*, *CYP27B1*	[40]

Search criteria are reported in Materials and Methods section.

## Data Availability

The original contributions presented in the study are included in the article. Further inquiries can be directed to the corresponding author.

## Supplementary Materials

The following supporting information can be downloaded at: https://www.mdpi.com/article/10.3390/genes16111311/s1, Table S1: List of the co-segregating variants with their key annotations.
